# Vitamin B Complex Treatment Attenuates Local Inflammation after Peripheral Nerve Injury

**DOI:** 10.3390/molecules24244615

**Published:** 2019-12-17

**Authors:** Adil Ehmedah, Predrag Nedeljkovic, Sanja Dacic, Jelena Repac, Biljana Draskovic Pavlovic, Dragana Vucevic, Sanja Pekovic, Biljana Bozic Nedeljkovic

**Affiliations:** 1 Institute of Physiology and Biochemistry “Ivan Djaja”, Faculty of Biology, University of Belgrade, 11000 Belgrade, Serbia; aozhe77@gmail.com (A.E.); sanjas@bio.bg.ac.rs (S.D.); jelenag@bio.bg.ac.rs (J.R.); 2 Institute for Orthopedic Surgery “Banjica”, 11000 Belgrade, Serbia; nedeljkovicpredrag@gmail.com; 3 Institute for Medical Research, Faculty of Medicine, Military Medical Academy, University of Defense in Belgrade, 11000 Belgrade, Serbia; biljadp@gmail.com (B.D.P.); draganavucevic@yahoo.com (D.V.); 4 Department of Neurobiology, Institute for Biological Research “Sinisa Stankovic”, National Institute of Republic of Serbia, University of Belgrade, 11060 Belgrade, Serbia; sanjapekovic@gmail.com

**Keywords:** peripheral nerve injury, neuroinflammation, regeneration, M1/M2 macrophages, calcium channels, vitamin B complex therapy

## Abstract

Peripheral nerve injury (PNI) leads to a series of cellular and molecular events necessary for axon regeneration and reinnervation of target tissues, among which inflammation is crucial for the orchestration of all these processes. Macrophage activation underlies the pathogenesis of PNI and is characterized by morphological/phenotype transformation from proinflammatory (M1) to an anti-inflammatory (M2) type with different functions in the inflammatory and reparative process. The aim of this study was to evaluate influence of the vitamin B (B1, B2, B3, B5, B6, and B12) complex on the process of neuroinflammation that is in part regulated by l-type Ca_V_1.2 calcium channels. A controlled transection of the motor branch of the femoral peripheral nerve was used as an experimental model. Animals were sacrificed after 1, 3, 7, and 14 injections of vitamin B complex. Isolated nerves were used for immunofluorescence analysis. Treatment with vitamin B complex decreased expression of proinflammatory and increased expression of anti-inflammatory cytokines, thus contributing to the resolution of neuroinflammation. In parallel, B vitamins decreased the number of M1 macrophages that expressed the Ca_V_1.2 channel, and increased the number of M2 macrophages that expressed this channel, suggesting their role in M1/M2 transition after PNI. In conclusion, B vitamins had the potential for treatment of neuroinflammation and neuroregeneration and thereby might be an effective therapy for PNI in humans.

## 1. Introduction

Injury to the peripheral nervous system (PNI) of the upper and lower limbs represents a serious issue in developed countries due to the increasing incidence associated with the modern lifestyle, characterized by high rate of traffic injuries, industrial traumatism, and injuries in the workplace. In Europe, the incidence of PNI is estimated at ~300,000 cases per year [1,2]. The major problem from socio-economic perspective is the fact that PNI commonly occurs in young male subjects at their most productive age, where actively serving military officers comprise a significant fraction of affected individuals. Concerning the low rate of recovery, this consequently leads to reduced working capabilities and overall life quality.

PNI triggers Wallerian degeneration, where neuroinflammation—mediated by series of cellular and molecular events—represents the most important stage for axon regeneration and reinnervation of target tissues [3]. Investigation of PNI pathophysiology revealed that Schwann cells and not the neurons are mainly affected by PNI. They are also suggested to be the primary mediators in triggering many of the events in Wallerian degeneration, while changes in their protein expression at the site of injury are supposed to be the key to axon regeneration [4]. Moreover, Schwann cells, together with inflammatory cells that infiltrate damaged tissue, represent crucial components of a series of neuroinflammatory events involved in injury and regeneration of the peripheral nerve. Among them, neutrophils are the first cell type that penetrates the injured nerve, followed by monocytes that differentiate to tissue macrophages, responsible for phagocytosis of myelin and tissue debris [5,6,7,8]. On the top of this, haematogenic macrophages, together with resident macrophages, enable the production of cytokines and neurotrophic factors necessary for activation of Schwann cells and extracellular matrix (ECM) remodeling, which is crucial for axon regeneration [4,9]. 

In general, macrophages are classified into two main subtypes: (1) M1 macrophages, which are active at inflammatory sites as primary phagocytic cells; and (2) M2 macrophages, which take over the tissue remodeling process after the inflammatory activity of M1 cells. According to this, M1 cells are known as classically activated or proinflammatory macrophages, while M2 cells are designated as alternatively activated or anti-inflammatory macrophages [10,11,12]. M1 activity assumes production of proinflammatory cytokines that can be neurotoxic to the regenerating axon, thus potentially amplifying the on-going process of neurodegeneration. To impede this potentially neurotoxic effect of M1 cells, the timely activation of reparatory M2 cells is crucial for initiation and execution of the cascade of neuroreparative events. M2 macrophages encompass a functionally diverse group of macrophages that are further divided into the M2a, M2b, M2c, and M2d subtypes [13,14,15]. 

The pro-healing activity of macrophages is further supported by hyper-proliferative, resident Schwann cells [16], which undergo a process of de-differentiation to the stage of pre-myelinating cells, releasing cytokines important for monocyte infiltration to the injury site [17]. Upon this, Schwann cells make novel contact with tissue macrophages, causing them to re-differentiate to the re-myelinating phenotype crucial for axonal regeneration. Further on, the interaction of macrophages with the newly-synthesized myelin can lead to resolution of inflammatory processes at the injured nerve. Thus, this bidirectional interaction between Schwann cells and macrophages is crucial for the tight control of peripheral nerve regeneration [18,19]. 

Processes underlying macrophage activation, such as alterations in morphology, proliferation, and production of proinflammatory mediators, are associated with calcium entry via the L type of voltage-dependent calcium channels (LVDCCs) [20,21,22]. PNI induces changes in the expression pattern of LVDCC isoform Ca_V_1.2, primarily through membrane potential regulation [23]. This process is also substantially modulated by soluble molecules—hormones, cytokines, and neurotransmitters [24]. Also, it has been shown that Ca^2+^ influx mediated by l-type VDCCs is necessary for normal myelination and facilitates axon–glial interaction during the first steps of myelin formation [25,26]. Their expression has been shown in Schwann cells as well [23,27]. Broad expression of l-type VDCCs and their involvement in the processes of inflammation and regeneration make them an interesting target for different therapeutic modalities.

For mechanical damage to the injured nerve, surgical treatment is the first therapeutic choice for most types of PNI. While being indispensable, surgical treatment requires additional adjuvant therapeutic modalities to maximize the recovery process [28]. Vitamins from the B group, separately or in different combinations, are used for the treatment of central and peripheral nervous system injuries, giving the best results in neuropathic pain reduction [29] and improvement of regenerative capacity [30]. Additionally, Altun and Kurutas [31] showed that injury to the sciatic nerve is accompanied by a lower level of vitamin B complex and vitamin B12 in homogenates of injured nerve after seven days, suggesting that supplementation of these vitamins would be beneficial for acceleration of nerve regeneration. Therefore, the aim of this study was to evaluate the influence of vitamin B (B1, B2, B3, B5, B6, and B12) complex therapy on the processes of neuroinflammation and neuroregeneration in the rat model of the femoral nerve motor branch injury. This assumes examining the effects of vitamin B complex therapy on pro- and anti-inflammatory cytokine production and phenotype profiles of macrophages (M1 and M2). Since these processes are partly regulated by Ca_V_1.2 subunit of LVDCCs, the potential role of this channel have been investigated. 

## 2. Results

### 2.1. Treatment with Vitamin B Complex Alters Macrophage Morphology after PNI

**Figure 1:**
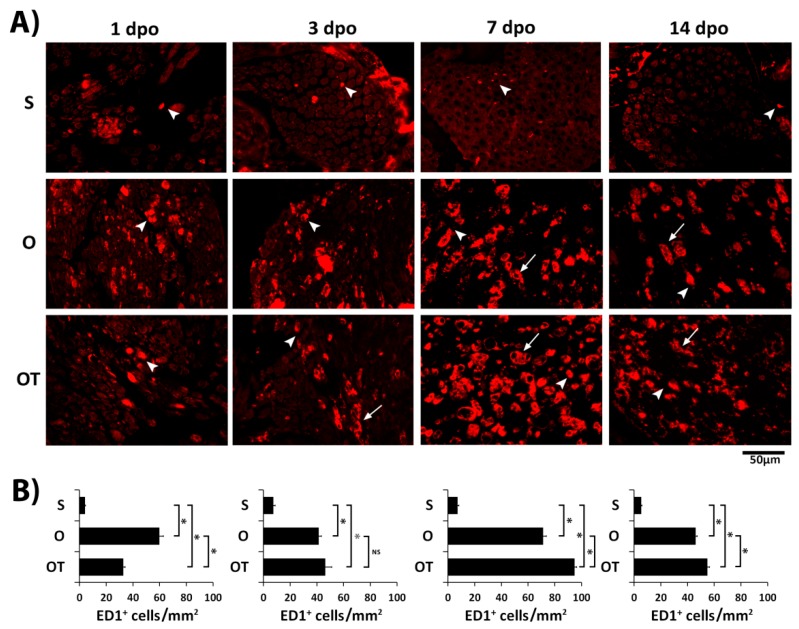
Effect of peripheral nerve injury (PNI) and treatment with B vitamins on macrophage morphology. Cross sections of femoral nerve obtained from the sham (S), operated (O, transection of motor branch and immediate reconstruction using termino–terminal anastomosis), and operated and treated with vitamin B complex (B1, B2, B3, B5, B6, and B12) (OT) groups were stained for ED1 (red) which is a common marker of activated macrophages. (**A**) The representative images showed morphological changes of ED1^+^ macrophages during the postoperative period (1, 3, 7, and 14 days) and after 1, 3, 7, and 14 injections of complex of B vitamins. Transition from the M1 (arrow heads) to M2 (arrows) morphology type in the O group is seen at day 7 and 14 post-injury. After treatment with B vitamins the appearance of M2 macrophages began after the third injection. Scale bar: 50 µm. (**B**) Total quantification of ED1-positive cells/mm^2^ from experimental groups is depicted in the graphs (black bars). The data are shown as the mean ± SEM of three independent experiments (three images/group/independent experiment were captured). Statistical analysis was performed using a two-sided Student’s *t*-test (* *p* < 0.05 vs. control, or vs. O group, as indicated at the graphs).

Macrophage activation underlies the pathogenesis of PNI and is characterized by morphological transformation. Also, depending on extracellular milieu they have capability to switch from proinflammatory to anti-inflammatory activation phenotype participating sequentially in both the induction and the resolution of inflammation [32]. Therefore, to estimate the impact of PNI and the effects of vitamin B complex treatment on macrophage activation, we first followed changes of macrophages morphology during the post-injury period (1, 3, 7, and 14 days post operation (dpo), Figure 1A). As a commonly utilized marker of activated macrophages we used the ED1 antibody (Table 1). The total number of ED1^+^ cells is presented in Figure 1B as a number of ED1^+^/mm^2^. In the sham-operated (S) group only a few ED1^+^ cells were detected and this number was not significantly changed at all investigated time points (Figure 1A,B). The number of ED1^+^ cells dramatically increased after PNI in the operated (O) group, peaking at the 7 dpo. Interestingly, at days 1 and 3 post-injury these ED1^+^ cells had a morphology resembling the M1 phenotype, while at 7 and 14 dpo most of them acquired the M2 phenotype. Treatment with B vitamins reduced the number of proinflammatory M1 macrophages at day 1 post-injury for 45% (Figure 1B), while starting from day 3 until 14 dpo enlarged the number of ED1^+^ macrophages with the “foamy” morphology of the M2 phenotype (Figure 1A). The effect of B vitamins was particularly pronounced at 7 dpo (the increase was 33% compared to the O group). These results indicated that PNI induced time-dependent changes in the macrophages morphology and transition from M1 proinflammatory to M2 regenerative type during the recovery period. Treatment with vitamin B complex accelerates this transition.

### 2.2. The Effect of Vitamin B Complex on Expression of Pro-Inflammatory Mediators after PNI

**Figure 2:**
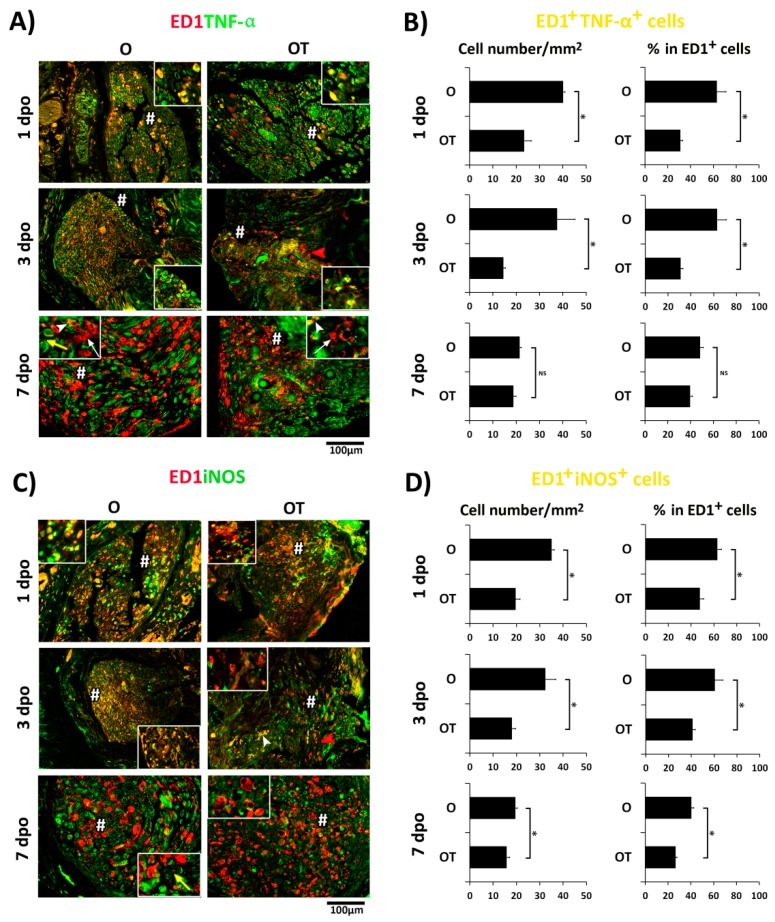
Treatment with B vitamins reduced PNI-induced expression of proinflammatory mediators in M1 macrophages. (**A**,**C**) Cross sections of femoral nerves obtained from the operated (O) and operated and treated with vitamin B complex (OT) groups were counterstained with (**A**) anti-TNF-α (M1 marker, green) and anti-ED1 (red) or with (**C**) anti-iNOS (M1 marker, green) and anti-ED1 (red) antibodies. The quantification of double-positive ED1^+^/TNF-α^+^ cells (**B**) and ED1^+^/iNOS ^+^ cells (**D**) is presented as number of double-positive cells/mm^2^ and the percentage of double-positive cells in the ED1^+^ cell population. The data are shown as the mean ± SEM of three independent experiments (three images/group/independent experiment were captured). Statistical analysis was performed using a two-sided Student’s *t*-test (* *p* < 0.05 OT vs. O group, as indicated at the graphs). At day 1 and 3 days post operation (dpo) ED1^+^/TNF-α^+^, as well as ED1^+^/iNOS^+^ macrophages in both the O and OT groups, had oval and round morphology and showed complete overlapping (yellow fluorescence)(inserts). Treatment with B vitamins reduced TNF-α and iNOS staining and the majority of macrophages were only ED1^+^ (red arrow head). At day 7 post-injury most of macrophages were only ED1^+^ and were polarized toward M2 type (white arrows, insert), while only a few ED1^+^/TNF-α^+^ (white arrow head, insert) macrophages were noticed. Some ED1^−^ cells (yellow arrows) that were both TNF-α^+^ and iNOS^+^ were also noticed. # indicates where the high magnification images in inserts are taken from. Scale bar: 100 µm. PNI: peripheral nerve injury; TNF: tumor necrosis factor; iNOS: inducible nitric oxide synthase.

Given that tumor necrosis factor alpha (TNF-α) and inducible nitric oxide synthase (iNOS) are mainly expressed marker proteins in M1 macrophages [10,11,12], we then investigated whether treatment with B vitamins would modulate expression profile of these cytokines in macrophages after PNI. At day 1 post-injury a huge number of ED1^+^/TNF-α^+^ (Figure 2A, insert) and ED1^+^/iNOS^+^ cells (Figure 2C, insert) (yellow) with round and oval shapes were widespread through the nerve slices of O group. Treatment with the cocktail of B vitamins reduced TNF-α and iNOS staining in ED1^+^ macrophages (Figure 2A,C, OT, inserts). The total number of ED1^+^/TNF-α^+^ cells and ED1^+^/iNOS^+^ cells is presented in Figure 2B,D, respectively, as a number of double-positive cells/mm^2^ and the percentage of double positive cells in the ED1^+^ cell population. A similar pattern of staining was detected at 3 dpo and after three treatments with B vitamins. At 7 dpo most of the ED1^+^ macrophages in both the O and OT group acquired a “foamy” morphology characteristic of M2 phenotype. However, they were not TNF-α and iNOS co-stained (Figure 2A,C (inserts)). Only those ED1^+^ cells which still had oval/round morphology were co-labeled with TNF-α and iNOS. Importantly, both TNF-α and iNOS staining was detected in some ED1^−^ cells (Figure 2, yellow arrows). Obtained results confirmed our previous observation that PNI induces expression of proinflammatory mediators in M1 macrophages, and that over time post-injury this M1 proinflammatory type of macrophages was replaced with the M2 type. Treatment with B vitamins significantly reduces the number of ED1^+^ macrophages expressing proinflammatory mediators, TNF-α, and iNOS, and in that way has an anti-inflammatory effect. These effects were the most pronounced at days 1 and 3 post-injury.

### 2.3. The Effect of Vitamin B Complex on Expression of Anti-Inflammatory Cytokines after PNI

**Figure 3:**
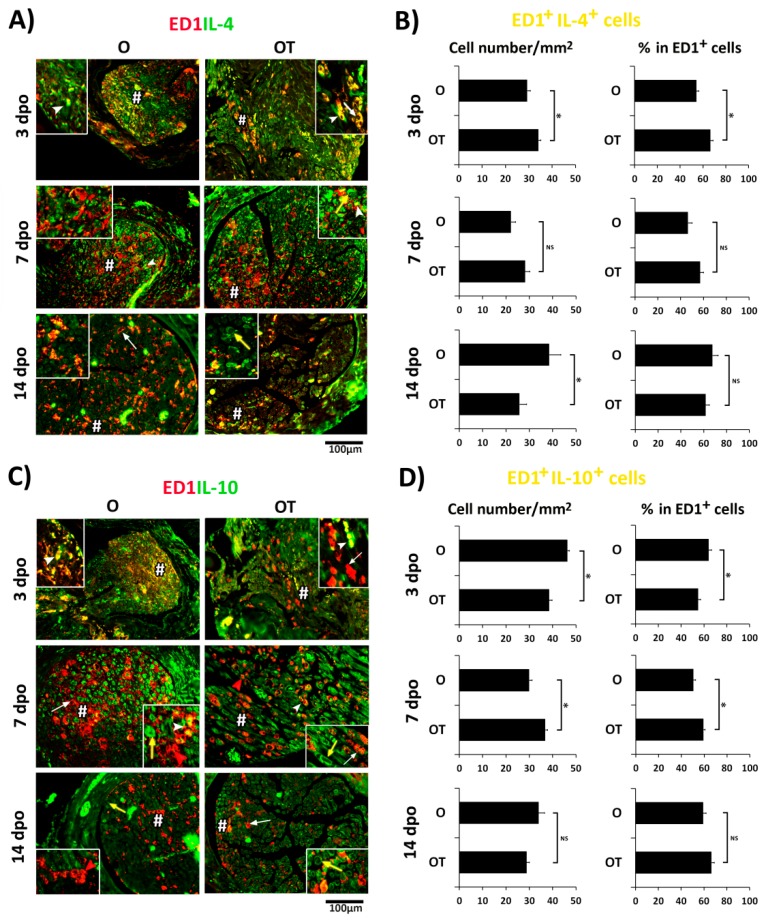
Effects of PNI and B vitamin treatment on expression of anti-inflammatory cytokines in M2 macrophages. (**A**,**C**) Cross sections of femoral nerve obtained from the operated (O) and operated and treated with vitamin B complex (OT) groups were counterstained with (**A**) anti-IL4 (M2a marker, green) and anti-ED1 (red) or with (**C**) anti-IL10 (M2c marker, green) and anti-ED1 (red) antibodies. The quantification of double-positive ED1^+^/IL-4^+^ cells (**B**) and ED1^+^/IL-10^+^ cells (**D**) is presented as number of double-positive cells/mm^2^ and the percentage of double positive cells in the ED1^+^ cell population. The data are shown as the mean ± SEM of three independent experiments (three images/group/independent experiment were captured). Statistical analysis was performed using a two-sided Student’s *t*-test (* *p* < 0.05 OT vs. O group, as indicated at the graphs). At day 3 post-injury ED1^+^/IL-4^+^ as well as ED1^+^/IL-10^+^ macrophages with oval and round morphology in both the O and OT groups showed complete overlapping (yellow fluorescence, white arrow head) (inserts). Treatment with B vitamins increased IL-4 immunoreactivity in “foamy” M2 macrophages (white arrow), while IL-10 staining was reduced and the majority of M2 macrophages were only ED1^+^ (white arrow). At 7 and 14 dpo IL-4 and IL-10 staining was seen in ED1^−^ cells in both groups (yellow arrows). M2 macrophages were void of IL-4 at day 7, but IL-4 was abundantly present at day 14 (white arrow). ED1^+^/IL-10^+^ M2 macrophages were seen at 7 dpo (white arrows), but were sparsely present at day 14. # indicates where the high magnification images in inserts are taken from. Scale bar: 100 µm. PNI: peripheral nerve injury; IL-4: interleukin -4; IL-10: interleukin-10.

Further, we wanted to determine whether treatment with vitamin B complex would enhance expression of anti-inflammatory cytokines, interleukin (IL)-4 and IL-10, in ED1^+^ macrophages (Figure 3). It is shown that the IL-4 cytokine is expressed in M2a, while the IL-10 is a marker of the M2c subtype of M2 macrophages [13,15,27,33]. At 3 dpo in the O group of animals complete overlapping (yellow) of IL-4 (green) and ED1 (red) staining was detected in macrophages with round and oval cell body (Figure 3A, insert). After treatment with B vitamins, macrophages in the injured nerve acquired a “foamy” morphology and were intensively labeled with anti-IL-4 antibody (Figure 3A, insert). Similarly, a huge number of ED1^+^/IL-10^+^ macrophages with a round and oval cell body (Figure 3C, insert) were detected in the O group. However, in the OT group large ED1^+^ macrophages were not labeled with anti-IL-10 antibody, and only those with small, round, and oval cell bodies (Figure 3C, insert) were ED1^+^/IL-10^+^ (Figure 3C and 3C(insert)). At 7 dpo in both the O and OT groups (Figure 3A and 3A(inserts)), most of the ED1^+^ cells had a “foamy” morphology and were IL-4 negative, although a few ED1^+^/IL-4^+^ macrophages with round and oval cell bodies were found. Interestingly, IL-4 expression was seen in some ED1^−^ cells as well. Similarly, at the same time point post-injury, abundant IL-10 expression was seen in some ED1^−^ cells in both O and OT groups. In the O group, ED1^+^ macrophages with “foamy” morphology did not express IL-10 and were pre-dominant compared to those with an oval morphology that were ED1^+^/IL-10^+^ co-stained (Figure 3C and 3C(insert)). In contrast, after treatment with complex of B vitamins the foamy macrophages were ED1^+^/IL-10^+^ (Figure 3C and 3C(insert)), while those with round and oval morphology were only ED1^+^. At 14 dpo the number of ED1^+^/IL-4^+^ cells, as well as ED1^+^/IL-10^+^ cells, was decreased after treatment with vitamin B complex, while the fractions of these cells in total ED1^+^ cell population were the same in the O and OT group. The total number of ED1^+^/IL-4^+^ cells and ED1^+^/IL-10^+^ cells is presented in Figure 3B,D, respectively, in terms of the number of double-positive cells/mm^2^ and the percentage of double-positive cells in the ED1^+^ cell population. Taken together, these results suggest that PNI and treatment with B vitamins cause the appearance of different subtypes of M2 macrophages.

### 2.4. Treatment with Vitamin B Complex Reduces Number M1 Macrophages Expressing the Ca_V_1.2 Channel after PNI

**Figure 4:**
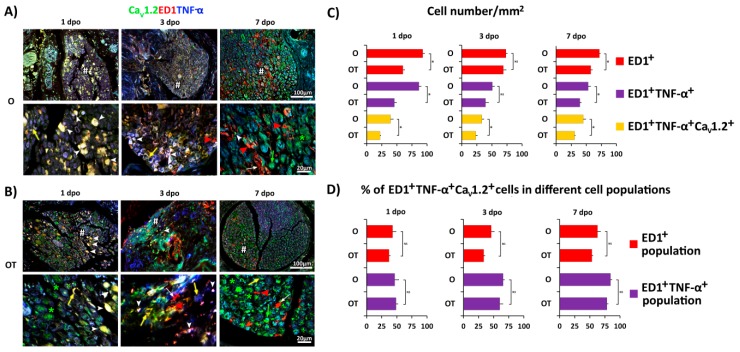
Treatment with vitamin B complex induces time-dependent changes of Ca_V_1.2 channel expression in M1 macrophages after PNI. To evaluate cellular distribution of the Ca_V_1.2 isoform of l-VDCCs (green), cross sections of femoral nerve obtained from the: (**A**) operated (O); and (**B**) operated and treated with vitamin B complex (OT) groups were counterstained with anti-TNF-α (M1 marker, blue) and anti-ED1 (red) antibodies. The quantification of single-, double-, and triple-positive cells is presented as number of ED1^+^ cells/mm^2^, ED1^+^/TNF-α^+^ cells/mm^2^, and ED1^+^/TNF-α^+^/Ca_V_1.2^+^ cells/mm^2^ (**C**), and as the percentage of triple -positive cells (ED1^+^/TNF-α^+^/Ca_V_1.2^+^ cells) in ED1^+^ and ED1^+^/TNF-α^+^ cell populations (**D**). The data are shown as the mean ± SEM of three independent experiments (three images/group/independent experiment were captured). Statistical analysis was performed using a two-sided Student’s *t*-test (* *p* < 0.05 OT vs. O group, as indicated at the graphs). Intensive Ca_V_1.2 staining, besides in M1 macrophages, was observed in axons (green asterisks) and in some ED1^−^ cells (yellow and green arrows) as well. ED1^+^/Ca_V_1.2^+^/TNF-α^+^ M1 macrophages are marked with a white arrowhead, ED1^+^ macrophages with oval/round morphology (M1 type) are marked with a red arrowhead, and “foamy” ED1^+^ macrophages (M2) are indicated with white arrows. # indicates where the high magnification micrographs are taken from. Scale bars: 20 µm and 100 µm. PNI: peripheral nerve injury; TNF: tumor necrosis factor.

Having in mind that Ca^2+^ influx mediated by l-type VDCCs is necessary for macrophage activation, alterations in morphology, and production of proinflammatory mediators [22], we next evaluated the effects of PNI and treatment with B vitamins on expression of the Ca_V_1.2 isoform of l-VDCCs in the injured peripheral femoral nerve. As shown in Figure 4A in the O group, expression of Ca_V_1.2 gradually decreases with time elapsed from PNI. The highest number of Ca_V_1.2^+^/ED1^+^/TNF-α^+^ was observed at day 1 post-injury (Figure 4C). At 7 dpo most of the ED1^+^ macrophages acquired a “foamy” morphology characteristic for the M2 phenotype and were Ca_V_1.2^−^/TNFα^−^. Treatment with complex of B vitamins (Figure 4B,C) significantly diminished the number of proinflammatory M1 macrophages (ED1^+^/TNF-α^+^) that co-express the Ca_V_1.2 isoform. However, the fractions of Ca_V_1.2^+^/ED1^+^/TNF-α^+^ cells in total macrophages (ED1^+^ cells) and M1 macrophages (ED1^+^/TNF-α^+^ cells) were the same in the O and OT group. Interestingly, concomitantly with down-regulation of Ca_V_1.2 expression in M1 macrophages, its expression was significantly up-regulated in axons and in some ED1^−^ cells (Figure 4A,B, higher magnification, green asterisks and yellow arrows, respectively). In summary, these results indicate that treatment with B vitamins reduced the number of M1 macrophages that express Ca_V_1.2 channel.

### 2.5. Treatment with Vitamin B Complex Alters Expression of Ca_V_1.2 Channel in M2 Macrophages after PNI

**Figure 5:**
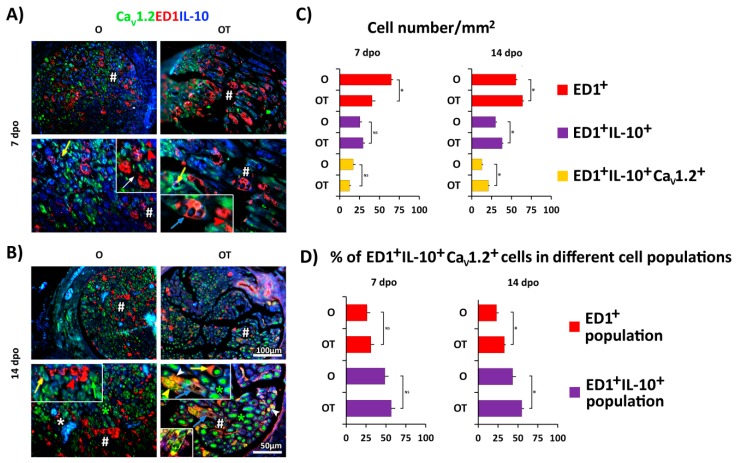
Treatment with vitamin B complex induces time-dependent changes in Ca_V_1.2 channel expression in M2 macrophages after PNI. To evaluate cellular distribution of the Ca_V_1.2 isoform of the L type of voltage-dependent calcium channels (l-VDCCs (green)), triple immunofluorescence staining of femoral nerve cross sections obtained from the operated (O) and operated and treated with vitamin B complex (OT) groups at (**A**) 7 dpo and (**B**) 14 dpo was performed. Anti-Ca_V_1.2 (green), anti-IL-10 (M2 marker, blue), and anti-ED1 (red) antibodies were used. The quantification of single, double, and triple-positive cells is presented as the number of ED1^+^ cells/mm^2^, ED1^+^/IL-10^+^ cells/mm^2^, and ED1^+^/IL-10^+^/Ca_V_1.2^+^ cells/mm^2^ (**C**) and as the percentage of triple-positive cells (ED1^+^/ IL-10^+^/Ca_V_1.2^+^ cells) in ED1^+^ and ED1^+^/IL-10^+^ cell populations (**D**). The data are shown as the mean ± SEM of three independent experiments (three images/group/independent experiment were captured). Statistical analysis was performed using a two-sided Student’s *t*-test (* *p* < 0.05 OT vs. O group, as indicated at the graphs). In the O group at both time-points (7 and 14 dpo) ED1^+^ macrophages with either oval or “foamy” morphology (inserts, red arrowhead and white arrows, respectively) were not co-stained with Ca_V_1.2 and IL-10. After treatment with 7 and particularly after 14 injections of B vitamins they were mostly ED1^+^/Ca_V_1.2^+^/IL-10^+^ (inserts, white arrowheads and blue arrows, respectively). At 14 dpo in the OT group some of the “foamy” macrophages were ED1^+^/Ca_V_1.2^+^/IL-10^−^ (yellow arrowheads), and these IL-10^−^ cells were ED1^+^/Arg-1^+^ (yellow, insert in the left lower corner). Intensive Ca_V_1.2 staining was seen in axons (green asterisks) and in some ED1^−^/IL-10^+^ cells (yellow and green arrows). # indicates where the high magnification micrographs are taken from. Scale bars: 50 µm and 100 µm. PNI: peripheral nerve injury; IL-10: interleukin-10.

Next, we examine the effects of PNI and treatment with B vitamins on Ca_V_1.2 expression in different subtypes of M2 macrophages. As shown in Figure 3, M2 type of macrophages were predominant type at day 7 and 14 post-injury and therefore we focused our attention on these two time-points (Figure 5A,B, respectively). At both time points, in the O group most of the ED1^+^ macrophages had a “foamy” morphology of the M2 type and were not co-stained with anti-Ca_V_1.2 antibody (Figure 5A and 5B(inserts)). The total number of ED1^+^/IL-10^+^/Ca_V_1.2^+^ cells is presented in Figure 5C. The fractions of Ca_V_1.2^+^/ED1^+^/IL-10^+^ cells in total macrophages (ED1^+^ cells) and M2 macrophages (ED1^+^/IL-10^+^ cells) were the same in O and OT group at 7 dpo. In contrast, in the group treated with B vitamins these macrophages with “foamy” morphology were ED1^+^/Ca_V_1.2^+^/IL-10^+^. At 14 dpo we detected Ca_V_1.2 expression in different types of macrophages. As shown in Figure 5B and 5B(insert) some of the macrophages had a more oval morphology and were ED1^+^/Ca_V_1.2^+^. Macrophages that had “foamy” morphology showed different IL-10 expression (Figure 5B insert in the left upper corner): some of them were ED1^+^/Ca_V_1.2^+^, but IL-10^−^ (yellow arrowhead), whereas others were ED1^+^/Ca_V_1.2^+^/IL-10^+^ (blue arrow). Interestingly, these “foamy” ED1^+^/IL-10^−^ cells were positive for arginase-1 (Arg-1, Figure 5B insert in the left lower corner), which is a considered a classic M2a marker [15,34]. As previously mentioned, the most intensive Ca_V_1.2 immunoreactivity was detected in axons (green asterisks) and some ED1^−^ IL-10^+^ cells (yellow arrows), and this was particularly extensive at 14 dpo in the OT group (Figure 5B and 5B(upper insert)). The fractions of Ca_V_1.2^+^/ED1^+^/IL-10^+^ cells in total macrophages (ED1^+^ cells) and M2 macrophages (ED1^+^/IL-10^+^ cells) were statistically higher in OT group at 14 dpo. These results indicate that Ca_V_1.2 has a time-dependent pattern of expression in different types of M2 macrophages. 

## 3. Discussion

Surgery represents the first therapeutic method of choice to treat most types of PNI, where direct microsurgical nerve repair or autologous nerve grafts are currently considered as gold standard treatments. Upon undergoing surgical reconstruction, the peripheral nerve has an innate capacity to induce the process of repair; however, the regeneration of motor and sensory functions often remains incomplete. Thus, the development of alternative repair strategies and treatments to complement well established surgical procedures is recognized as highly needed, and therefore represents a very attractive area of research [35].

Among variety of proposed adjuvant therapeutic modalities related to peripheral nerve regeneration, in this work we focused our attention on vitamins of the B complex (B1, B2, B3, B5, B6, B12) as possible candidates to treat PNI, due to their infinite renewability and amenability to molecular manipulation. Namely, vitamins of the B group act as coenzymes in a substantial fraction of enzymatic processes and play key interacting roles in a majority of cellular functions, thus being important for normal functioning of the nervous system as well [36]. Importantly, due to their well-recognized positive effects on both the central and peripheral nervous system, they are already often used in the treatment of various pathological conditions [37,38,39]. Vitamin B12 has been shown in vivo to be the most effective of all B vitamins in peripheral nerve regeneration. The positive effect is manifested in several ways: B12 enhances neuronal survival and axonal outgrowth after trauma by activation of Erk1/2 and Akt protein kinases [40], facilitates axonal sprouting from the proximal to the distal part of the injured nerve [41], and has a positive effect on Schwann cell proliferation during regeneration [41]. It also improves myelination of axons, i.e., by increasing the diameter of the myelin sheath, and accelerating and enhancing axonal maturation. These positive effects of vitamin B12 are due to its metabolic activity. In addition to these effects, Fujii et al. [42] demonstrated that vitamins B1, B6 as well as B 12 increase the rate of impulse delivery through the nerve (by reduction of axon degeneration in rat acrylamide-induced neuropathy) and enhance the outgrowth of regenerating axons. Some data indicate that supplementation of B12 vitamin and other B vitamins in the acute period after peripheral nerve injury may be beneficial for the acceleration of nerve regeneration [31]. Additionally, there is evidence that vitamin B12 in combination with dexamethasone promotes peripheral nerve repair [43]. All presented findings provide new insight into the role of vitamins of the B complex and support the investigation of each B vitamin or their combinations for further investigation of their effects as a potential treatment of peripheral nerve injury.

As a reaction to PNI, a strong proinflammatory immune response, mediated by Schwann cells, resident macrophages, and fibroblasts, is triggered as a consequence of blood-borne monocytes infiltrating the damaged nerve only two to three days upon injury [44]. To enable adequate propagation of neuroreparation/neuroregeneration, this first response to PNI needs to be modulated by the anti-inflammatory process, mediated by the same type of cells at the injury site. Clearly, these antagonistic immune activities invoke mediator cells to undergo functional and phenotypic transformation. 

Macrophages have long been held as potent immune effector cells with well-established roles in both, the tissue homeostasis and injuries, e.g., in the promotion of the tissue injury initiation and progression and also wound healing improvement and tissue remodeling in various pathological conditions [45,46]. Noticeably, mounting evidence from a number of different in vivo and in vitro studies has generally demonstrated that identification of the activated macrophage states together with macrophage M1 to M2 polarization targeting (or vice versa) might serve as novel therapeutic strategies to treat different pathological conditions [47,48,49], such as PNI.

Time-dependent changes in macrophage morphology, characterized by the transition from round shaped, smaller M1 (proinflammatory) to a “foamy” shaped, larger M2 (anti-inflammatory) phenotype was confirmed by our results during the recovery period after PNI. Interestingly, our results also indicate that the vitamin B complex treatment accelerates this M1 to M2 transition, and, as we recently published [30], was accompanied with improved recovery of the motor nerve and locomotor performances in rats. The observed change in macrophage morphology during recovery from PNI, along with its acceleration in response to the vitamin B complex treatment, was confirmed by the cytokine production of the involved macrophages. In this context, our results demonstrated that PNI induces the expression of TNF-α and iNOS in macrophages, which then represent the proinflammatory M1 phenotype, being in line with the well-established role of activated M1 macrophages at the injury site during Wallerian degeneration [44]. Further on, the replacement of the type M1 with M2 macrophages during the post-injury period was evidenced by the production of IL-4 and IL-10. A number of factors can affect the process of macrophage type/phenotype transition, including local cytokine milieu at the injury site, molded by the neuronal and Schwann cell activity [18]. Treatment with B vitamin complex reduced the number of M1 macrophages, limiting their effects up to 3 dpo. Concerning this, we conclude that the treatment with the vitamin B complex expresses an anti-inflammatory effect, thus limiting the damage of the injured nerve by shortening the transition period from the indispensable inflammation immediately after PNI to the process of neuroreparation, mediated by M2 macrophages. IL-10 is an anti-inflammatory cytokine whose up-regulation is shown from 7 days up to 28 days after injury in the distal segment of the nerve [50,51]. Its well-known role is in modulation of proinflammatory cytokine expression and axonal plasticity [52]. Given that macrophages are the main cell type that expresses this cytokine [50], increased expression of IL-10 in “foamy” M2c macrophages that we detected after treatment with B vitamins, therefore, may contribute to resolution of inflammation induced by femoral nerve injury and promotion of nerve repair.

The versatile effects of the investigated vitamin B complex treatment should be considered as an important result, keeping in mind that most of the compounds that reduce neuroinflammation act only as M1 macrophage inhibitors [53]. Most importantly, our data show that M1/M2 polarization balance after PNI can be rapidly induced and completely reversed by the vitamin B complex treatment during early period of the recovery, whereas underlying cellular signaling pathways need to be elucidated in upcoming studies. Overall, macrophage polarization plasticity provides a basis for macrophage-centered therapeutic strategies as an alternative repair approach to complement the surgery after PNI. Concerning this, in our future work, the exact cellular pathways underlying the macrophage subset differentiation, induced by the combination of B vitamins, which we used in this study, will be thoroughly investigated and discussed. 

Recently, results of several studies indicated that the Schwann cells most likely interact with macrophages to support their function in peripheral nerve injury, probably via expressing several ligands that are known to interact with receptors expressed by macrophages, and that Schwann cells may regulate M1/M2 transition [18]. Moreover, it was shown that Schwann cells secrete classic M2-associated cytokines and are potent inducers of M2-phenotypes in macrophages, and that these macrophages promote axonal outgrowth [18]. Accordingly, in our study we have noted up-regulation of anti-inflammatory cytokines, IL-4 and IL-10, in ED1^−^ cells with Schwann-cell-like morphology at day 7 post-injury, and to lesser extent at 14 dpo, which was significantly potentiated after treatment with vitamin B complex. This was accompanied with increased appearance of “foamy” M2 macrophages co-expressing IL-4 and/or IL-10. Interestingly, at 7 dpo we also noted a PNI-induced increase of the proinflammatory mediators TNF-α and iNOS, with immunostaining in some ED1^−^ cells resembling Schwann-cell-like morphology, particularly at the site of nerve injury. Similarly, Dubový et al. [54] suggested that such a simultaneous induction of proinflammatory and anti-inflammatory cytokines in Schwann cells after PNI is responsible for maintaining a balance in the inflammatory reaction of Schwann cells and in promoting axonal growth. However, one could not exclude that the macrophage–Schwann cell interaction operates vice versa since macrophages are shown to regulate Schwann cell maturation after nerve injury [19]. 

Given that calcium entry via LVDCCs is associated with changes in macrophage morphology, proliferation, and production of pro- and anti-inflammatory mediators [20,21,22], is necessary for normal myelination, and facilitates axon–glial interaction during the myelin formation [25,26], we further characterized their involvement in the observed effects of PNI and treatment with B vitamins. Specifically, we focused our attention to Ca^2+^ signaling via Ca_V_1.2 LVDCC isoform that appeared as “a new player” in the regulation of cell activation, proliferation, survival, and cytokine production of immune cells [55,56,57] and Schwann cells as well [23,27]. Since our data point to M1 toward M2 polarization during recovery period after femoral nerve injury, which is facilitated after administration of B vitamins, we investigated whether this phenotypic switch includes changes in the expression of the Ca_V_1.2. Indeed, the most intensive Ca_V_1.2 immunoreactivity was noted in ED1^+^/TNF-α^+^ M1 macrophages at day 1 post-injury and gradually declined with time elapsed from PNI and was concomitant with transition from M1 toward M2 type of macrophages. Similar involvement of Ca_V_1.2 in controlling microglial proinflammatory activity was detected in a rat model of *N*-methyl-d-aspartate-induced hippocampal neurodegeneration [22]. However, data concerning their role in neuroinflammatory processes after PNI are still obscure. Considering the effect of B vitamins on Ca_V_1.2 expression in macrophages, it is important to note that applied treatment reduces the number of proinflammatory M1 (Ca_V_1.2^+^/ED1^+^/TNF-α^+^) macrophages, but increases Ca_V_1.2 abundance in regenerative M2 (M2a and M2c) macrophages. These results suggest that investigated channel may have implications in nerve protection and repair. In addition, we demonstrated remarkable Ca_V_1.2 up-regulation in myelinated and to less extent in non-myelinated Schwann cells implying their potential role in activity of these cells after the PNI. The most pronounced expression of Ca_V_1.2 was detected in axons of all investigated groups. Similarly, high levels of immunostaining of l-type Ca^2+^ channels were found in odontoblast cell bodies and their processes, in fibroblast cell bodies, and in Schwann cells, as well as in unmyelinated and myelinated axons in root nerves and proximal branches in coronal pulp [23]. However, immunostaining of these LVDCCs was shown to be transiently down-regulated in response to injury.

## 4. Materials and Methods

### 4.1. Experimental Protocol

In total, 48 adult male Albino Oxford (AO) rats, weighing between 250 and 300 g, were used throughout the study. Experimental animals were randomly divided into three groups (containing 16 animals per group). The first group comprised “operated animals” (O), in which transection of the femoral nerve motor branch was performed with immediate reconstruction using a technique of termino-terminal anastomosis. The second group (OT) included animals that were surgically treated in the same way but were additionally receiving vitamin B complex therapy. The third group included “sham operated” animals (S), which underwent the same procedure (dissection of the motor branch of the femoral nerve), but without transection of the nerve. All groups were additionally divided into sub-groups (four per group) that were sacrificed 1, 3, 7, and 14 days post-operation (dpo). Before and during the experiment, all animals were kept in the same environmental conditions (laboratory temperature 23 ± 2 °C, humidity between 50% and 60%, 12  h/12  h light/dark cycle with lights on at 07:00 h, free availability of water and food). All animal experiments were approved by the Ethics Review Committee for Animal Experimentation of Military Medical Academy and Ministry of Agriculture and Environmental Protection Republic of Serbia, Veterinary Directorate No. 323-07-7363/2014-05/5.

#### Surgery

Controlled transection of the peripheral nerve is a well-described model for the examination of peripheral nerve regeneration [58]. Animals were anesthetized by intraperitoneal application of ketamine (50 mg/kg; Ketalar, Eczacibasi, Turkey) and xylazine (5 mg/kg; Rompun, Bayer, Turkey). Following anesthesia, the animals from all investigated groups (S, O, and OT) were appropriately positioned for identification of the femoral nerve motor branch on the rat left hind paw by skin incision in the left groin and femoral region, under aseptic conditions (as described in [30]). In all groups of animals (S, O, and OT), the motor branch was identified just before entry into the quadricep muscle. Further, in animals from O and OT group, using microscope magnification, transection of the branch was done and immediate reconstruction performed using a 10.0 non-absorbable suture in the form of termino-terminal anastomosis. The skin was sutured using a 4.0 absorbable suture (Peters Surgical, Paris, France). All animals used in the experiments survived the surgical procedure and were subjected to the same set of analyses. At the appropriate time point the rats were sacrificed by intravenous injection of ketamine/xylazine at a lethal dose. All procedures were done in accordance with the Guide for the Care and Use of Laboratory Animals. Motor branches of the femoral nerve (both reconstructed and intact contralateral) were isolated and further used for immunofluorescence analysis.

### 4.2. Treatment Protocol

For the investigation of vitamin B complex treatment, ampoules (2 mL) of Beviplex (Beviplex^®^, Galenika a.d. Belgrade, Serbia), each containing B1 (40 mg), B2 (4 mg), B3 (100 mg), B5 (10 mg), B6 (8 mg), and B12 (4 µg), were used. The given dose was 1.85 mL/kg/day. The vitamin B complex was injected intraperitoneally immediately (15 min) after the operation and then every 24 h from the day of the operation until the day of sacrifice. Operated but untreated animals (O), were intraperitoneally injected with the same volume of physiological solution. 

### 4.3. Paraffin Tissue Preparation

The isolated nerves were prepared for immunohistochemistry in the Laboratory for Pathohistology and Cytology HistoLab, Belgrade, by the following the procedure: 

The isolated nerve samples underwent the fixation procedure in the 10% formaldehyde solution to preserve the tissue morphology and antigenicity of target molecules on the dissected nerve. Prior to the addition of melted paraffin wax, the isolated tissue underwent a series of dehydration steps at room temperature (RT): (1) 3 × 30 min in 70% ethanol; (2) 3 × 30 min in 90% ethanol; (3) 3 × 30 min in 100% ethanol; and (4) 3 × 30 min in xylene. Following dehydration, the tissue was immerged into the melted paraffin wax at 58 °C. Microtome sectioning of the paraffin-embedded tissue was next done at a thickness of 5 µm. Sections were then incubated at 56 °C in water bath, mounted onto histological slides pre-coated with gelatin for better tissue adhesion, and dried overnight at RT.

### 4.4. Immunofluorescence Staining

Immunofluorescence (IF) staining was used for protein localization on tissue slides. For immunofluorescent staining, the fluorescent-dye conjugated secondary antibody which binds to the unlabeled primary antibody was used. Except for the incubation with primary antibody, which was performed at a temperature of 4 °C, the IF staining procedure was done at RT. All the solutions were prepared in 0.01 M PBS, pH 7.4, which was also used for washing agent after certain steps. Double IF staining proceeded according to the following steps:

Deparaffinization and rehydration: Microscope slides with paraffin-embedded sections were deparaffinized and rinsed in xylene 1, xylene 2, absolute alcohol, 95% alcohol, 70% alcohol, and distilled water, for 5 min in each solution. Antigen retrieval: Antigenic epitope unmasking was done by boiling microscope slides in 0.01 M sodium citrate buffer, pH 6, for 8 min at 99%–100 °C, followed by cooling at RT for 30 min and 3 × 5 min PBS washing. Blocking solution: After the washing step, microscope slides were incubated for 60 min in 5% blocking serum (originating from the same species as the secondary antibody) to prevent nonspecific binding of the secondary antibody. To enable membrane permeabilization, 0.5% Triton X-100 detergent was added to the blocking serum. Primary antibody, diluted in PBS, was applied onto slides and incubated overnight at 4 °C temperature. Next day, slides were washed out 3 × 5 min in PBS. Secondary antibody, diluted in PBS, was applied onto slides, where it specifically binds to the present primary antibody. Slides were next washed for 3 × 5 min in PBS. In the case of double or triple IF staining, the steps starting from the incubation in the blocking serum were repeated for the next markers. The primary and secondary antibodies used for IF labeling are indicated in the Table 1. After incubation with the last secondary antibody slides were washed 6 × 5 min in PBS and mounted with Mowiol (Calbiochem, Millipore, Germany). After drying overnight, slides were ready for viewing under the microscope. As a staining control, microscope slides that underwent the same IF procedure, but without the primary antibody application, were used. 

### 4.5. Digital Image Processing

The images of the prepared nerve sections were acquired using Carl Zeiss Axiovert fluorescent microscope, equipped with the AxioCam monochromatic camera (Axio Observer Microscope Z1, ZEISS, Gottingen, Germany), at the magnifications of 20×, 40×, 63×, and 100× and saved in .tiff format. To capture images at 63× and 100× magnification ApoTome software was used. Co-localization on the obtained fluorescent images was done using AxioVision Rel. 4.6 program, which represents a standard part of the Zeiss Axiovert microscope equipment, and then assembled and labeled in Photoshop CS6 (Adobe Systems). The quantification of single, double, or triple-positive cells from experimental groups (S, O, OT) was performed for each time point (1, 3, 7, 14 dpo), and obtained from three independent experiments. High resolution digital images (600 pixels/inch) captured at 40× magnification (three images/group/independent experiment) were used for cells counting. The total number of single, double, or triple-positive cells was counted manually by two independent observers using ImageJ open-source platform (National Institutes of Health, USA; http://imagej.nih.gov/ij/download.html) with ImageJ cell-counter plugin (https://imagej.nih.gov/ij/plugins/cell-counter.html) and Adobe Photoshop Creative Cloud (Version 14.0). Additionally, the percentage of double or triple-positive cells in some investigated cells populations was calculated and presented.

### 4.6. Statistical Analysis

For statistical comparison between two experimental groups a two-sided Student’s *t*-test was performed and a value of *p* < 0.05 or less was considered significant. Values were shown as mean values with standard error (SEM).

## 5. Conclusions

In conclusion, we report for the first time that treatment with a complex of B (B1, B2, B3, B5, B6, and B12) vitamins could effectively promote PNI-induced M1 to M2 macrophage polarization and suppress inflammatory response by reducing expression of proinflammatory and up-regulation of anti-inflammatory cytokines. Moreover, our findings point to the potential involvement of the Ca_V_1.2 subunit of the l type of VDCCs in the processes of inflammation after PNI. Treatment with B vitamins increases Ca_V_1.2 abundance in M2 macrophages, suggesting their mediating role in improving recovery of the injured nerve. Thus, our study exhibited that B vitamins, owing to their pleiotropic effects, had the potential for treatment of neuroinflammation and neuroregeneration, and can be considered as a promising adjuvant therapy of PNI in humans, which remains to be further explored.

## Figures and Tables

**Table 1:** List of primary and secondary antibodies used for immunofluorescence labeling.

Antibody	Source	Dilution	Company
anti-Ca_V_1.2	rabbit	1:200	Sigma-Aldrich, Munich, Germany
anti-CD68 (ED1)	mouse	1:100	Abcam, Cambridge, MA, USA,
anti-TNF-α	goat	1:100	Santa Cruz Biotechnology, CA, USA
anti-iNOS	rabbit	1:100	Santa Cruz Biotechnology, CA, USA
anti-IL-4	rabbit	1:100	Santa Cruz Biotechnology, CA, USA
anti-IL-10	goat	1:100	Santa Cruz Biotechnology, CA, USA
anti-Arg-1	rabbit	1:200	Sigma-Aldrich, Munich, Germany
anti-rabbit anti-IgG Alexa Fluor 488	donkey	1:200	Invitrogen, Carlsbad, CA, USA
anti-mouse anti-IgG Alexa Fluor 555	donkey	1:200	Invitrogen, Carlsbad, CA, USA
anti-goat anti-IgG Alexa Fluor 350	donkey	1:200	Invitrogen, Carlsbad, CA, USA
anti-goat anti-IgG Alexa Fluor 488	donkey	1:200	Invitrogen, Carlsbad, CA, USA
